# Supplementary material for: Negativity drives online news consumption
Source: Nat Hum Behav. 2023 Mar 16;7(5):812–22. doi: 10.1038/s41562-023-01538-4 (PMC10202797; doi:10.1038/s41562-023-01538-4)
Supplement: Supplementary file 1 — Supplementary Tables 1–25 and Figs. 1–11. [file 41562_2023_1538_MOESM1_ESM.pdf]

nature human behaviour

Registered Report https://doi.org/10.1038/s41562-023-01538-4

# Negativity drives online news consumption

In the format provided by the authors and unedited

# Supplementary Information

## Contents


**A Descriptive statistics** **3**

**B Frequency of positive and negative dictionary words** **6**

**C Estimation results** **7**

**D Robustness checks** **8**

D.1 Analysis with alternative sentiment dictionaries . . . . . . . . . . . . . . . . . . . . . 8

D.2 Negation handling . . . . . . . . . . . . . . . . . . . . . . . . . . . . . . . . . . . . 11

D.3 Alternative text complexity measures . . . . . . . . . . . . . . . . . . . . . . . . . . 13

D.4 Quadratic effects . . . . . . . . . . . . . . . . . . . . . . . . . . . . . . . . . . . . . 16

D.5 Analysis of positive-only and negative-only headlines . . . . . . . . . . . . . . . . . 18

D.6 Analysis of image RCTs . . . . . . . . . . . . . . . . . . . . . . . . . . . . . . . . . 19

D.7 Regression analysis based on sentiment . . . . . . . . . . . . . . . . . . . . . . . . . 20

D.8 Regression analysis with log-transformation . . . . . . . . . . . . . . . . . . . . . . . 21

**E The role of moralized language as a moderator** **22**

**F Negativity effects across topics** **25**

F.1 Procedure for topic modeling . . . . . . . . . . . . . . . . . . . . . . . . . . . . . . . 25

F.2 Overview of generated topics . . . . . . . . . . . . . . . . . . . . . . . . . . . . . . . 26

F.3 Validation of topic model . . . . . . . . . . . . . . . . . . . . . . . . . . . . . . . . . 28

F.4 Regression analysis with topic controls . . . . . . . . . . . . . . . . . . . . . . . . . 29

F.5 Regression analysis with topic-specific negativity effects . . . . . . . . . . . . . . . . 30

**G Extension to discrete emotions** 31

G.1 Frequency of emotional words from NRC emotion lexicon . . . . . . . . . . . . . 31

G.2 Estimation results for discrete emotions . . . . . . . . . . . . . . . . . . . . . . . . . . 33

G.3 Analysis with topics and emotions . . . . . . . . . . . . . . . . . . . . . . . . . . . . . 34

**H User studies to validate dictionary approach** 35

**I Analysis across all basic emotions and higher-order emotions** 39

I.1 Analysis for basic emotions . . . . . . . . . . . . . . . . . . . . . . . . . . . . . . . . 39

I.2 Analysis for bipolar emotion pairs . . . . . . . . . . . . . . . . . . . . . . . . . . . . 42

I.3 Analysis for emotional dyads . . . . . . . . . . . . . . . . . . . . . . . . . . . . . . . 45

# A Descriptive statistics

Supplementary Table 1 reports descriptive statistics on the confirmatory dataset from Registered Report Stage 2. *Clicks* denotes the raw number of clicks that a give headline received. *Impressions* denotes the number of Upworthy user that were assigned a given headline. The *CTR* gives the click-through rate, that is, the ratio of clicks per impression. The distribution of the CTR is positively skewed (skewness: $2.43$) indicating that the mass of the distribution is concentrated at lower values.

Word counts for sentiment and emotional words (i. e., before $z$-standardization) are as follows. $Positive$ and $Negative$ describe the percentage of words in each headline that belong to the positive and negative word lists in the LIWC dictionary, respectively. $Anger$, $Anticipation$, $Disgust$, $Fear$, $Joy$, $Sadness$, $Surprise$, and $Trust$ are the scores for the 8 basic emotions calculated based on the NRC emotion lexicon. These scores range between zero and one and sum up to one across the basic emotions.

Further controls are as follows. *Length* is the number of words in each headline. $Complexity$ gives the Gunning-Fog index score for each headline. $PlatformAge$ is the age of the platform, that is, the number of days since the first ever Upworthy experiment being conducted. For example, a headline with a value of 100 for $PlatformAge$ was published 100 days after the first Upworthy story.

We further elaborate the dependence structure between positive and negative words. Supplementary Figure 1 visualizes the density of the proportion of positive and negative words in headlines. The figure shows that a large density in the bottom-left corner, representing headlines where both positive and negative words from the LIWC are absent (36.4 %). In addition, we find that headlines often contain exclusively positive (29.6 %) or exclusively negative words (20.9 %). This motivates later one of our robustness checks where we perform a regression analysis based on headlines with positive-only and negative-only dictionary words. Only 13.0 % of all headlines contain both positive and negative words. The overall correlation (Pearson's $r$) between the proportion

of positive and negative words in headlines is $-0.074$ ($p < 0.001$).

| Variable | Mean | Median | Min | Max | Std. Dev. |
|---|---|---|---|---|---|
| Outcomes | | | | | |
| *Clicks* | 51.735 | 37.000 | 0.000 | 1091.000 | 51.882 |
| *Impressions* | 3824.001 | 3182.000 | 1.000 | 42,850.000 | 2081.582 |
| *CTR* (click-through rate) | 0.014 | 0.011 | 0.000 | 0.149 | 0.011 |
| Dictionary-based variables | | | | | |
| $Positive$ | 0.037 | 0.000 | 0.000 | 0.500 | 0.051 |
| $Negative$ | 0.028 | 0.000 | 0.000 | 0.444 | 0.044 |
| $Anger$ | 0.092 | 0.000 | 0.000 | 1.000 | 0.178 |
| $Anticipation$ | 0.162 | 0.000 | 0.000 | 1.000 | 0.241 |
| $Disgust$ | 0.067 | 0.000 | 0.000 | 1.000 | 0.155 |
| $Fear$ | 0.134 | 0.000 | 0.000 | 1.000 | 0.214 |
| $Joy$ | 0.144 | 0.000 | 0.000 | 1.000 | 0.214 |
| $Sadness$ | 0.103 | 0.000 | 0.000 | 1.000 | 0.191 |
| $Surprise$ | 0.063 | 0.000 | 0.000 | 1.000 | 0.150 |
| $Trust$ | 0.235 | 0.143 | 0.000 | 1.000 | 0.301 |
| Control variables | | | | | |
| *Length* | 14.965 | 15.000 | 1.000 | 37.000 | 3.112 |
| *Complexity* | 8.409 | 8.200 | 0.400 | 40.400 | 3.657 |
| *PlatformAge* | 487.726 | 539.000 | 0.000 | 825.000 | 205.904 |

Supplementary Table 1: Descriptive statistics.

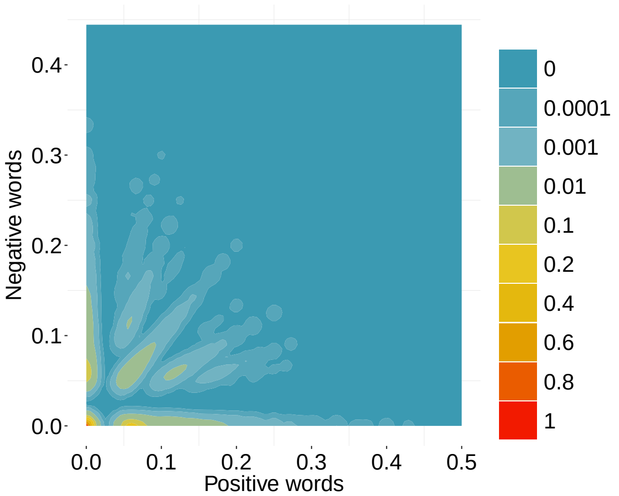


Supplementary Figure 1: Dependency between positive and negative words. The density plot shows the relationship between the proportion of positive and negative words in headlines. Red (blue) corresponds to a higher (lower) density. Density is normalized to go from 0 to 1.

# B Frequency of positive and negative dictionary words

A list of the most frequent dictionary words in our dataset is given in Supplementary Table 2 (positive and negative words).

| *Positive* | | *Negative* | |
|---|---|---|---|
| Word | Frequency | Word | Frequency |
| love | 980 | wrong | 728 |
| pretty | 746 | bad | 588 |
| beautiful | 645 | awful | 363 |
| truth | 505 | hate | 300 |
| hilarious | 480 | war | 294 |
| amazing | 448 | worst | 245 |
| save | 418 | sick | 236 |
| funny | 397 | fight | 229 |
| awesome | 386 | scary | 225 |
| care | 365 | hell | 213 |

Supplementary Table 2: Top 10 most frequent positive and negative words, as defined by the LIWC dictionary, in our sample.

# C Estimation results

Detailed estimation results for all model parameters are reported for our main analysis examining the role of positive and negative words (Supplementary Table 3).

| | Coef | Lower CI | Upper CI | $P$-value |
|---|---|---|---|---|
| $Positive$ | $-0.008$ | $-0.010$ | $-0.006$ | $< 0.001$ |
| $Negative$ | $0.015$ | $0.013$ | $0.018$ | $< 0.001$ |
| $Length$ | $0.041$ | $0.038$ | $0.043$ | $< 0.001$ |
| $Complexity$ | $-0.004$ | $-0.006$ | $-0.001$ | $< 0.001$ |
| $PlatformAge$ | $-0.309$ | $-0.323$ | $-0.295$ | $< 0.001$ |
| (Intercept) | $-4.475$ | $-4.490$ | $-4.461$ | $< 0.001$ |

Observations: 53,699

Supplementary Table 3: Regression model explaining click-through rate based on positive and negative words in headlines. Reported are standardized coefficient estimates and $99\,\%$ CIs. $P$-values are calculated using two-sided $z$-tests. Experiment-specific intercepts (i. e., random effects) are included. $N = 53{,}669$ headlines were examined over $12{,}448$ RCTs.

# D Robustness checks

## D.1 Analysis with alternative sentiment dictionaries

In our main analysis, we use positive and negative words from the LIWC. We now validate our results based on alternative word lists. Specifically, we compare the estimates from the following dictionaries:

1. **LIWC** (main paper) [4] We compute scores for positive and negative words (i. e., $Positive$ and $Negative$) using the built-in dictionary from Linguistic Inquiry and Word Count (LIWC). The estimation results are those from the main paper.

2. **NRC** [1] The NRC emotion lexicon comprises 181,820 English words that are classified into positive and negative words. We use the implementation from the `sentimentr` package to calculate the proportion of positive words ($Positive_{\text{NRC}}$) and negative words ($Negative_{\text{NRC}}$) in headlines.

3. **SentiStrength.** SentiStrength is a sentiment dictionary that was primarily developed for short social media texts [5]. SentiStrength returns two integer scores, namely $Negative_{\text{SS}} \in [-5, -1]$ for the negative sentiment and $Positive_{\text{SS}} \in [1, 5]$ for the positive sentiment. Note that, in SentiStrength, lower values of $Negative_{\text{SS}}$ correspond to more negative sentiment. We thus multiply $Negative_{\text{SS}}$ by $-1$ to facilitate comparability to the other dictionaries.

Based on the above dictionaries, we then fitted separate regression models, one for each sentiment score. We again used $z$-standardization for better comparisons. Overall, the estimated 99 % confidence intervals (CIs) from all models are in good agreement (Supplementary Table 4 and Supplementary Figure 2). In particular, the regression models suggest that negative words increase click-through rates. This finding is consistent across all considered dictionaries.

| | Coef | Lower CI | Upper CI | $P$-value |
|---|---|---|---|---|
| LIWC | | | | |
| *Positive* | $-0.008$ | $-0.010$ | $-0.006$ | $< 0.001$ |
| *Negative* | $0.015$ | $0.013$ | $0.018$ | $< 0.001$ |
| *Length* | $0.041$ | $0.038$ | $0.043$ | $< 0.001$ |
| *Complexity* | $-0.004$ | $-0.006$ | $-0.001$ | $< 0.001$ |
| NRC | | | | |
| *Positive* | $-0.008$ | $-0.011$ | $-0.006$ | $< 0.001$ |
| *Negative* | $0.007$ | $0.005$ | $0.010$ | $< 0.001$ |
| *Length* | $0.040$ | $0.037$ | $0.042$ | $< 0.001$ |
| *Complexity* | $-0.004$ | $-0.006$ | $-0.001$ | $< 0.001$ |
| SENTISTRENGTH | | | | |
| *Positive* | $0.002$ | $0.000$ | $0.005$ | $0.007$ |
| *Negative* | $0.023$ | $0.020$ | $0.025$ | $< 0.001$ |
| *Length* | $0.039$ | $0.037$ | $0.041$ | $< 0.001$ |
| *Complexity* | $-0.005$ | $-0.008$ | $-0.003$ | $< 0.001$ |

Observations: $53{,}699$

Supplementary Table 4: Regression models comparing the effects of emotional words on the click-through rate across different sentiment dictionaries. Reported are standardized coefficient estimates and $99\,\%$ CIs. $P$-values are calculated using two-sided $z$-tests. Experiment-specific intercepts (i. e., random effects) are included. $N = 53{,}669$ headlines were examined over $12{,}448$ RCTs.

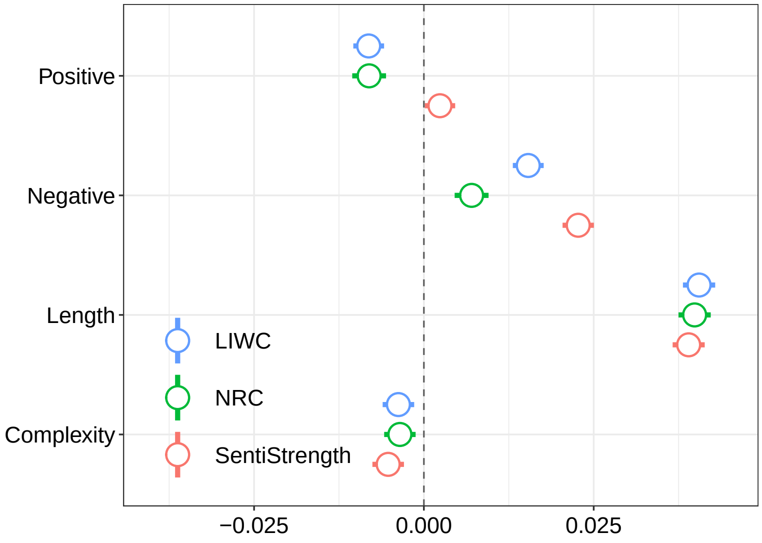


Supplementary Figure 2: Comparison showing that the effect of negative words on the click-through rate is robust across different sentiment dictionaries. Shown are the estimated standardized coefficients (circles) for positive and negative words and for further controls. The error bars correspond to the 99 % confidence intervals (CIs). The variable *PlatformAge* is included in the model during estimation but not shown for better readability. $N = 53{,}669$ headlines were examined over $12{,}448$ RCTs. Full estimation results are in Supplementary Table 4.

## D.2 Negation handling

Our main analysis accounts for negations by counting all words in the neighborhood of a negation word (e. g., “not,” “no”) as belonging to the opposite word list. In our analysis, the neighborhood (i. e., the so-called negation scope) was set to 3 words after the negation. As a robustness check, we experiment with an alternative neighborhood of 5 words before and 2 words after the negation. Here, the same list of negations as in the main paper is used. We then compare the coefficient estimates for the two different approaches to negation handling. Overall, we find high agreement for positive and negative words (Supplementary Table 5 and Supplementary Figure 3). In fact, all 99 % confidence intervals (CIs) overlap and, hence, yield similar results.

| | Coef | Lower CI | Upper CI | $P$-value |
|---|---|---|---|---|
| MAIN | | | | |
| *Positive* | −0.008 | −0.010 | −0.006 | < 0.001 |
| *Negative* | 0.015 | 0.013 | 0.018 | < 0.001 |
| *Length* | 0.041 | 0.038 | 0.043 | < 0.001 |
| *Complexity* | −0.004 | −0.006 | −0.001 | < 0.001 |
| ALTERNATIVE | | | | |
| *Positive* | −0.009 | −0.012 | −0.007 | < 0.001 |
| *Negative* | 0.017 | 0.014 | 0.019 | < 0.001 |
| *Length* | 0.040 | 0.038 | 0.043 | < 0.001 |
| *Complexity* | −0.004 | −0.006 | −0.001 | < 0.001 |

Observations: 53,699

Supplementary Table 5: Regression models comparing the effects of emotional words on the click-through rate across two different approaches to negation handling. The “main” approach uses a neighborhood of 3 words after the negation. The “alternative” approach uses a neighborhood of 5 words before and 2 words after the negation. Reported are standardized coefficient estimates and 99 % CIs. $P$-values are calculated using two-sided $z$-tests. Experiment-specific intercepts (i. e., random effects) are included. $N = 53{,}669$ headlines were examined over 12,448 RCTs.

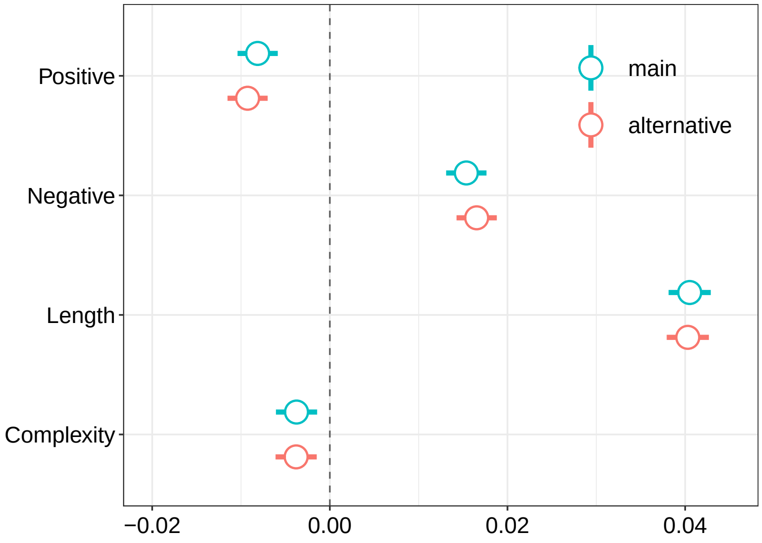


Supplementary Figure 3: Comparison of the effects of emotional words across two different approaches to negation handling. The "main" approach uses a neighborhood of 3 words after the negation. The "alternative" approach uses a neighborhood of 5 words before and 2 words after the negation. Shown are the estimated standardized coefficients (circles) for positive and negative words and for further controls. The error bars correspond to the 99 % confidence intervals (CIs). The variable *PlatformAge* is included in the model during estimation but not shown for better readability. $N = 53{,}669$ headlines were examined over $12{,}448$ RCTs. Full estimation results are in Supplementary Table 5.

## D.3 Alternative text complexity measures

The results in the main analysis use the Gunning-Fog Index as a measure of text complexity. As a robustness check, we calculate alternative text complexity measures and compare the estimates. Here, we use the implementation from the `quanteda` package (details: https://quanteda.io/reference/textstat_readability.html) to calculate the following text complexity measures:

1. **Gunning-Fog Index (Fog)** estimates the years of formal education necessary for a person to understand a text upon reading it for the first time. It is given by $0.4 \times (ASL + 100 \times n_{\text{wsy}\geq 3}/n_{\text{w}})$, where $ASL$ is the average sentence length (number of words), $n_{\text{w}}$ is the total number of words, and $n_{\text{wsy}\geq 3}$ is the number of words with three syllables or more. Larger values indicate greater text complexity.

2. **Automated Readability Index (ARI)** estimates an approximate representation of the US grade level needed to comprehend the text. Mathematically, it is computed via $0.5 \times ASL + 4.71AWL - 21.34$, where $ASL$ is the average sentence length (number of words), and $AWL$ is the average word length (number of characters). Larger values indicate greater complexity.

3. **Flesch's Reading Ease Score (Flesch)** is designed to rank how difficult a text in English is to understand. Formally, it is given by $206.835 - (1.015 \times ASL) - 84.6 \times (n_{\text{sy}}/n_{\text{w}})$, where $ASL$ is the average sentence length (number of words), $n_{\text{w}}$ is the total number of words, and $n_{\text{sy}}$ is the number of syllables. Flesch's Reading Ease Score is different from the other scores here in that larger values indicate *lower* text complexity.

4. **Average Word Syllables (AWL)** measures the average word syllables in a text. It is formalized as $n_{\text{sy}}/n_w$, where $n_{\text{w}}$ is the total number of words and $n_{\text{sy}}$ is the number of syllables. Larger values indicate greater complexity.

We fitted separate regression models, one for each text complexity score. We again used $z$-

standardization for better comparisons. Overall, we find that the different text complexity measures do not seem to be a reliable predictor of clicking rates, but we do find that the negativity bias is robust regardless of the text complexity measure used (Supplementary Table 6 and Supplementary Figure 4). Note that the coefficient for Flesch's Reading Ease Score points into the opposite direction because of its reverse interpretation (i. e., a larger value indicates *lower* complexity).

| | Coef | Lower CI | Upper CI | $P$-value |
|---|---|---|---|---|
| FOG | | | | |
| *Positive* | $-0.008$ | $-0.010$ | $-0.006$ | $< 0.001$ |
| *Negative* | $0.015$ | $0.013$ | $0.018$ | $< 0.001$ |
| *Length* | $0.041$ | $0.038$ | $0.043$ | $< 0.001$ |
| *Complexity* | $-0.004$ | $-0.006$ | $-0.001$ | $< 0.001$ |
| FLESCH | | | | |
| *Positive* | $-0.008$ | $-0.011$ | $-0.006$ | $< 0.001$ |
| *Negative* | $0.015$ | $0.013$ | $0.018$ | $< 0.001$ |
| *Length* | $0.040$ | $0.038$ | $0.043$ | $< 0.001$ |
| *Complexity* | $0.001$ | $-0.001$ | $0.004$ | $0.186$ |
| ARI | | | | |
| *Positive* | $-0.009$ | $-0.011$ | $-0.006$ | $< 0.001$ |
| *Negative* | $0.015$ | $0.013$ | $0.017$ | $< 0.001$ |
| *Length* | $0.041$ | $0.039$ | $0.043$ | $< 0.001$ |
| *Complexity* | $0.002$ | $0.000$ | $0.005$ | $0.009$ |
| AWL | | | | |
| *Positive* | $-0.008$ | $-0.011$ | $-0.006$ | $< 0.001$ |
| *Negative* | $0.015$ | $0.013$ | $0.018$ | $< 0.001$ |
| *Length* | $0.040$ | $0.038$ | $0.043$ | $< 0.001$ |
| *Complexity* | $-0.001$ | $-0.004$ | $0.001$ | $0.262$ |

Observations: 53,699

Supplementary Table 6: Regression models comparing the effects of emotional words on the click-through rate across four different approaches to measure text complexity. Larger values indicate higher text complexity for Fog, ARI, AWL and lower text complexity for Flesch. When correcting for the different interpretations and thus the opposite signs, the coefficients are in good agreement. Reported are standardized coefficient estimates and $99\,\%$ CIs. $P$-values are calculated using two-sided $z$-tests.Experiment-specific intercepts (i. e., random effects) are included. $N = 53{,}669$ headlines were examined over $12{,}448$ RCTs.

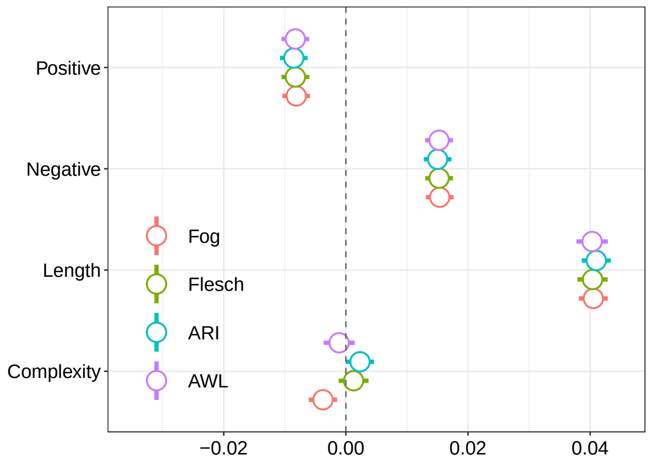


Supplementary Figure 4: Regression estimates for different measures of text complexity. Larger values indicate higher text complexity for Fog, ARI, AWL and lower text complexity for Flesch. When correcting for the different interpretations and thus the opposite signs, the coefficients are in good agreement. Shown are the estimated standardized coefficients (circles) for positive and negative words and for further controls. The error bars correspond to the 99 % confidence intervals (CIs). The variable *PlatformAge* is included in the model during estimation but not shown for better readability. $N = 53{,}669$ headlines were examined over $12{,}448$ RCTs. Full estimation results are in Supplementary Table 6.

## D.4 Quadratic effects

We extended our models to include quadratic effects for dictionary variables, that is, for the $Positive$ and $Negative$ variables (Supplementary Table 7 and Supplementary Figure 5). We find a negative and statistically significant quadratic effect for negative words. The quadratic effect of positive words is not statistically significant. All direct effects are still statistically significant. This result supports the robustness of our main analysis.

| | Coef | Lower CI | Upper CI | $P$-value |
|---|---|---|---|---|
| $Positive$ | $-0.005$ | $-0.008$ | $-0.002$ | $< 0.001$ |
| $Negative$ | $0.026$ | $0.023$ | $0.029$ | $< 0.001$ |
| $Positive^2$ | $-0.002$ | $-0.003$ | $-0.001$ | $< 0.001$ |
| $Negative^2$ | $-0.006$ | $-0.007$ | $-0.005$ | $< 0.001$ |
| $Length$ | $0.039$ | $0.036$ | $0.041$ | $< 0.001$ |
| $Complexity$ | $-0.004$ | $-0.006$ | $-0.001$ | $< 0.001$ |
| $PlatformAge$ | $-0.309$ | $-0.323$ | $-0.295$ | $< 0.001$ |
| (Intercept) | $-4.467$ | $-4.482$ | $-4.453$ | $< 0.001$ |

Observations: $53{,}699$

Supplementary Table 7: Regression results with quadratic effects for the word count variables. The dependent variable is the click-through rate. Reported are standardized coefficient estimates and $99\,\%$ CIs. $P$-values are calculated using two-sided $z$-tests.Experiment-specific intercepts (i. e., random effects) are included. $N = 53{,}669$ headlines were examined over $12{,}448$ RCTs.

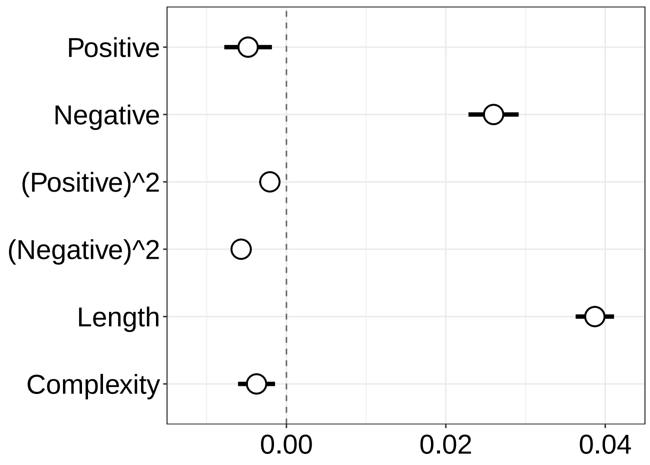


Supplementary Figure 5: The effect of positive and negative words in news headlines on the click-through rate while controlling for quadratic effects in the word count variables. Shown are the estimated standardized coefficients (circles) for positive and negative words and for further controls. The error bars correspond to the 99 % confidence intervals (CIs). The variable *PlatformAge* is included in the model during estimation but not shown for better readability. $N = 53{,}669$ headlines were examined over $12{,}448$ RCTs. Full estimation results are in Supplementary Table 7.

## D.5 Analysis of positive-only and negative-only headlines

In some cases, headlines might contain both positive and negative language. In our main paper, these headlines were coded as containing both positive and negative words and then used for estimation. However, headlines with a combination of both positive and negative language may be perceived differently by users than a positive-only headline or a negative-only headline. Hence, we repeated the analysis from the main paper but removed headlines where both positive and negative words were simultaneously present. As such, we end up with all headlines that exclusively include either positive or negative words, that is, positive-only and negative-only headlines. This led to a sample of 46,649 headlines from 12,189 RCTs.

Overall, we find that the negativity bias is robust even when dropping headlines that contained both positive and negative language.

| | Coef | Lower CI | Upper CI | $P$-value |
|---|---|---|---|---|
| $Positive$ | $-0.011$ | $-0.014$ | $-0.008$ | $< 0.001$ |
| $Negative$ | $0.013$ | $0.010$ | $0.016$ | $< 0.001$ |
| $Length$ | $0.042$ | $0.039$ | $0.044$ | $< 0.001$ |
| $Complexity$ | $-0.004$ | $-0.006$ | $-0.001$ | $< 0.001$ |
| $PlatformAge$ | $-0.307$ | $-0.321$ | $-0.292$ | $< 0.001$ |
| (Intercept) | $-4.489$ | $-4.503$ | $-4.474$ | $< 0.001$ |

Observations: 46,649

Supplementary Table 8: Regression results excluding positive and negative mixed headlines. The dependent variable is the click-through rate. Reported are standardized coefficient estimates and $99\,\%$ CIs. $P$-values are calculated using two-sided $z$-tests.Experiment-specific intercepts (i. e., random effects) are included. $N = 46{,}649$ headlines were examined over $12{,}189$ RCTs.

## D.6 Analysis of image RCTs

In addition to A/B testing headlines, Upworthy also A/B tested the images that were paired with each story. Most experiments tested either headlines or images, but there are occasions on which a headline RCT and an image RCT overlap. From the data in the Upworthy Research Archive, researchers can see which RCTs included an image test, but cannot see what images consisted of. We thus reran our main analyses excluding all headline RCTs that also contained image RCTs. This left 50,072 headlines in 111,373 RCTs. Overall, we find that the negativity bias is robust even when dropping headline RCTs that contained image RCTs also.

| | Coef | Lower CI | Upper CI | $P$-value |
|---|---|---|---|---|
| $Positive$ | $-0.008$ | $-0.010$ | $-0.006$ | $< 0.001$ |
| $Negative$ | $0.015$ | $0.013$ | $0.018$ | $< 0.001$ |
| $Length$ | $0.041$ | $0.039$ | $0.044$ | $< 0.001$ |
| $Complexity$ | $-0.003$ | $-0.005$ | $0.000$ | 0.005 |
| $PlatformAge$ | $-0.321$ | $-0.335$ | $-0.306$ | $< 0.001$ |
| (Intercept) | $-4.471$ | $-4.486$ | $-4.456$ | $< 0.001$ |

Observations: 50,072

Supplementary Table 9: Regression results for RCTs without image variations. The dependent variable is the click-through rate. Reported are standardized coefficient estimates and 99 % CIs. $P$-values are calculated using two-sided $z$-tests. Experiment-specific intercepts (i. e., random effects) are included. $N = 50{,}072$ headlines were examined over 11,373 RCTs.

## D.7 Regression analysis based on sentiment

We repeated the regression analysis based on a single sentiment score (as opposed two separate variables for positive and negativity). For this, we computed a single sentiment score, which is given by the net difference between the proportion of positive words and the proportion of negative words. Formally, this is given by $Sentiment = Positive - Negative$). We then estimated the model with the new sentiment variable but kept all other controls. The coefficient is negative and statistically significant ($p < 0.001$), implying that a positive sentiment decreases click-through rate while a negative sentiment increases click-through rate. This is consistent with the findings from our main analysis.

| | Coef | Lower CI | Upper CI | $P$-value |
|---|---|---|---|---|
| $Sentiment$ | −0.017 | −0.019 | −0.015 | < 0.001 |
| $Length$ | 0.040 | 0.037 | 0.042 | < 0.001 |
| $Complexity$ | −0.003 | −0.006 | −0.001 | < 0.001 |
| $PlatformAge$ | −0.310 | −0.324 | −0.296 | < 0.001 |
| (Intercept) | −4.475 | −4.490 | −4.461 | < 0.001 |

Observations: 53,699

Supplementary Table 10: Regression model explaining click-through rate based on the difference between the proportion of positive and negative words in headlines ($Sentiment$). Reported are standardized coefficient estimates and 99 % CIs. $P$-values are calculated using two-sided $z$-tests. Experiment-specific intercepts (i. e., random effects) are included. $N = 53{,}669$ headlines were examined over 12,448 RCTs.

## D.8 Regression analysis with log-transformation

*Note: The following robustness check was added during Stage 2 of the Registered Report (upon a reviewer's request). It was not part of the original planned analyses from Stage 1.*

The distribution of the CTR is positively skewed (skewness: $2.43$) indicating that the mass of the distribution is concentrated at lower values. As a robustness check, we estimated a regression model with the log-transformed CTR as the dependent variable (Supplementary Table 11). We find strong negativity effects consistent with those in the main analysis.

| | Coef | Lower CI | Upper CI | $P$-value |
|---|---|---|---|---|
| $Positive$ | $-0.011$ | $-0.016$ | $-0.006$ | $< 0.001$ |
| $Negative$ | $0.022$ | $0.016$ | $0.027$ | $< 0.001$ |
| $Length$ | $0.051$ | $0.045$ | $0.056$ | $< 0.001$ |
| $Complexity$ | $0.003$ | $-0.003$ | $0.008$ | $0.210$ |
| $PlatformAge$ | $-0.315$ | $-0.329$ | $-0.301$ | $< 0.001$ |
| (Intercept) | $-4.540$ | $-4.554$ | $-4.525$ | $< 0.001$ |

Observations: 53,659

Supplementary Table 11: Regression model explaining the log-transformed click-through rate. The dependent variable is $\log(CTR)$. Headlines that received zero clicks (40) are omitted due to log transformation. Reported are standardized coefficient estimates and $99\,\%$ CIs. $P$-values are calculated using two-sided $t$-tests. Experiment-specific intercepts (i. e., random effects) are included. $N = 53{,}669$ headlines were examined over $12{,}448$ RCTs.

# E The role of moralized language as a moderator

We investigated moralized language as a possible moderator of negativity in driving the click-through rate. This is motivated by previous research, whereby moral-emotional expressions have been found to play an important role in the diffusion of moralized online content [6]. We thus investigate the role of such moral words in moderating the effect of negative words on online news consumption. Analogous to Brady et al. [6], we extract the number of moral words in each headline using a dictionary containing 411 moral words, first presented in [7].

We extend the regression models from our main analysis with interaction terms between the moral word count and the proportion of positive and negative words. In addition, we include the proportion of moral words separately as a regressor. The results (Supplementary Table 12 and Supplementary Figure 6) show that moralized language decreases the click-through rate in online news. We found a negative and statistically significant direct effect of moralized language on click-through rate (coef: $-0.024$, SE = $0.001$, $z = -17.067$, $p < 0.001$, CI = [-0.028, -0.020]) and negative and statistically significant effects for the interactions between the proportion of moral words and the proportion of positive (coef: $-0.006$, SE = $0.001$, $z = -5.321$, $p < 0.001$, CI = [-0.010, -0.003]) and negative words (coef: $-0.007$, SE = $0.001$, z = $-7.048$, $p < 0.001$, CI = [-0.010, -0.005]). More importantly, even when controlling for a moderating role of moralized language, we find strong negativity effects consistent with those in the main analysis.

| | Coef | Lower CI | Upper CI | $P$-value |
|---|---|---|---|---|
| $MoralWords \times Positive$ | $-0.006$ | $-0.010$ | $-0.003$ | $< 0.001$ |
| $MoralWords \times Negative$ | $-0.007$ | $-0.010$ | $-0.005$ | $< 0.001$ |
| $MoralWords$ | $-0.024$ | $-0.028$ | $-0.021$ | $< 0.001$ |
| $Positive$ | $-0.004$ | $-0.007$ | $-0.002$ | $< 0.001$ |
| $Negative$ | 0.023 | 0.021 | 0.026 | $< 0.001$ |
| $Length$ | 0.042 | 0.040 | 0.044 | $< 0.001$ |
| $Complexity$ | $-0.004$ | $-0.006$ | $-0.002$ | $< 0.001$ |
| $PlatformAge$ | $-0.310$ | $-0.324$ | $-0.296$ | $< 0.001$ |
| (Intercept) | $-4.463$ | $-4.478$ | $-4.449$ | $< 0.001$ |

Observations: 53,659

Supplementary Table 12: Regression analysis with moralized language as a potential moderator for the effect of positive and negative words on click-through rate. Reported are standardized coefficient estimates and 99 % CIs. $P$-values are calculated using two-sided $z$-tests. Experiment-specific intercepts (i. e., random effects) are included. $N = 53{,}669$ headlines were examined over 12,448 RCTs.

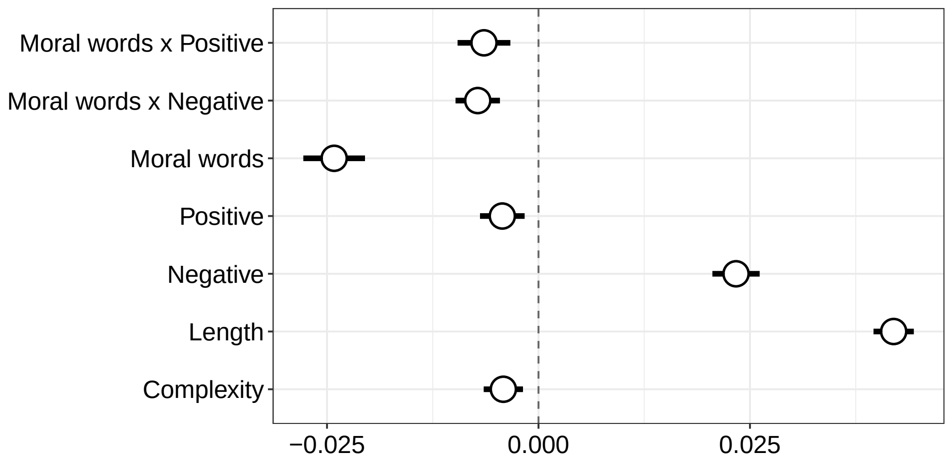


Supplementary Figure 6: Regression analysis with moralized language as a potential moderator for the effect of positive and negative words on click-through rate. Shown are the estimated standardized coefficients (circles). The error bars correspond to the 99 % confidence intervals (CIs). The variable *PlatformAge* is included in the model during estimation but not shown for better readability. $N = 53{,}669$ headlines were examined over $12{,}448$ RCTs. Full estimation results are in Supplementary Table 12.

# F Negativity effects across topics

We used an unsupervised machine learning framework to infer the distribution of news topics in the data through a bottom-up procedure. The benefit of using machine learning is that no assumptions are made ex ante with regard to the covered topics. Our machine learning approach is further regarded as superior to conventional topic modeling (i. e., latent Dirichlet analysis) with short texts [8]. Using the extracted topics, we can extend the regression from our main analysis to capture between-topic heterogeneity.

We applied the unsupervised machine learning framework to the exploratory sample from Registered Report Stage 1. This was done to train—and evaluate—the topic model. We then applied the identified topic model here in Stage 2 to map news headlines from the confirmatory sample onto the existing topics. In doing so, we obtain topic labels for each headline using the same categorization as in our pre-registration phase.

## F.1 Procedure for topic modeling

Our unsupervised machine learning framework proceeds in 4 steps. (1) We treat each RCT as corresponding to a single topic. Therefore, the headline variations from each RCT are concatenated to form a single document. (2) We encode the preprocessing document through a document embedding model [9]. (3) We apply $k$-means clustering to the document embeddings, thereby yielding $k$ different topic clusters. (4) We assign names to the topic cluster using a systematic procedure. For this, we manually inspect characteristic words and a number of sample headlines for each cluster. To retrieve the most characteristic words, we first concatenate all headlines belonging to a cluster into a single document and then apply stemming and stop-word removal. We then take the highest-ranking words according to the term frequency–inverse document frequency (tf-idf) statistic. Informed by these, names for each topic are assigned.

## F.2 Overview of generated topics

We apply the aforementioned machine learning framework to the Upworthy dataset. Upon manual inspection, the number of clusters was set to $k = 8$. This value was found to provide a suitable balance between sufficient granularity while maintaining interpretability. In particular, this value cluster headlines by overall themes (and not by individual news events). When producing topic names, we found that two of the eight clusters were better represented using a single topic name and were therefore merged. The resulting 7 clusters and summary statistics are reported in Supplementary Table 13. The summary statistics reveal that stories about people's lives are most common (29.8 %), followed by news about "Life" (16.5 %). Stories related to "Economy & Government" and other specific societal issues, such as "Woman Rights & Feminism" and "LGBT," were also frequent. Exemplary headlines for each topic are listed in Supplementary Table 14.

| | Topic name | Relative frequency | Characteristic words |
|---|---|---|---|
| 1 | Entertainment | 13.54% | peopl, watch, get, stewart, black, talk, jon, white, ask, comedian |
| 2 | Government & Economy | 11.99% | peopl, get, america, make, wage, us, minimum, food, work, like |
| 3 | LGBT | 4.62% | gay, peopl, straight, marriag, lesbian, guy, like, ask, get, way |
| 4 | Life | 16.47% | peopl, make, like, thing, world, know, video, get, see, life |
| 5 | Parenting & School | 10.99% | kid, girl, school, like, get, teacher, littl, teen, make, children |
| 6 | People | 29.80% | peopl, thing, make, like, get, say, guy, see, know, realli |
| 7 | Women Rights & Feminism | 12.59% | women, woman, feminist, like, think, girl, look, guy, get, know |

Supplementary Table 13: Summary statistics for the different topics embedded in the news stories from the exploratory sample ($N = 11{,}109$ headlines). Reported are also characteristic words of each topic defined as the top-10 words (stemmed) with regard to the tf-idf statistics.

| | Topic name | Sample Headlines |
|---|---|---|
| 1 | Entertainment | “The NFL May Get A Lot Of Things Wrong, But This Former Player Is 100% Right In His Rant On Spanking” |
| | | “Bill Nye Points Out The Biggest Problem With Modern Astrology” |
| | | “I’m Not A Conspiracy Theorist But Learning About Movie Ratings Has Me Reaching For The Tin Foil” |
| 2 | Government & Economy | “Mr. President, I’m Not Mad. I’m Just Disappointed. No, Wait. I’m A Little Mad Too.” |
| | | “Meet The Unmanned Drones Built To Fight Poverty Instead Of People” |
| | | “So That’s What Hard Working Government Employees Look Like? (Pssst...Can We Send This To Congress?) |
| 3 | LGBT | “Marriage In France Just Got A Lot Gayer” |
| | | “I Have A Really Secret Way To Help Protect Gay Kids From Bullying” |
| | | “Lets Have The Sexuality Talk And Clear 10 Things Up” |
| 4 | Life | “Why People Risk Their Lives for For People They’ve Never Met” |
| | | “A Newly Launched Camera Is Exposing Some Of Our Worst Parts” |
| | | “ Finally, An Approachable Guide To Crappy Arguments We See On The Internet. Every. Day.” |
| 5 | Parenting & School | “Want To Raise A Genius? Introduce Her To Bob Dylan.” |
| | | ”It’s Amazing What People Can Do When They Expect Their Children To Live Past Kindergarten” |
| | | “ Band Geeks Think They’re Smarter Than The Rest Of Us. Turns Out, They’re Right.” |
| 6 | People | “She Grew Up With Privilege – And She Knows How To Use It” |
| | | “It Broke Her Heart Seeing Her Daughter’s Facebook Page, Asking For Someone To Please Be Her Friend” |
| | | “Have You Ever Heard ’Don’t Act Like A Typical Tourist’? Here’s Why.” |
| 7 | Women Rights & Feminism | “Sexual Objectification: What it is, Why It’s Damaging, And How We Can Change It” |
| | | “A Tampon Commercial That Shows Just How Confusing Actual Tampon Commercials Are” |
| | | “Calling Girls This Word May Seem Harmless — But Why Are Boys Never Called It?” |

Supplementary Table 14: Examples of headlines assigned to the seven topics.

## F.3 Validation of topic model

Next, we validated our topic modeling approach by conducting a user study. Similar validations are also used in other research [10]. Specifically, we followed best-practice for validating topic models by implementing a *topic intrusion* test [11]. This test allows us to validate that participants were better than chance at categorizing headlines as belonging to a certain topic in accordance with our topic model. Participants ($n = 10$) recruited from the NYU subject pool were native speakers, provided informed consent, and and were granted .5 research credit hours for their participation. Participants were asked to read a random subset of 70 headlines. Participants were also shown four possible topic categories—the correct topic category and 3 other topics—from which they were asked to identify which category the headline belonged to. Participants answered 51.1 % of trials correctly. This is significantly above chance which would amount to having 25 % of the trials answered correctly ($\chi^2 = 249.61$, $p < 0.01$). The user study thus confirms that the topic model generates meaningful representations. The breakdown of correct answers per topic are listed below.

| Topic | Percent Correct |
|---|---|
| Entertainment | 45.0% |
| Government & Economy | 51.5% |
| LGTB | 67.0% |
| Life | 49.5% |
| Parenting & School | 43.0% |
| People | 25.3% |
| Women Rights & Feminism | 76.0% |

Supplementary Table 15: Percent correct from human validators in the Topic Intrusion Task, broken down by topic.

We considered the use of a word intrusion test [11] but eventually discarded this. Word intrusion tests for a small within-topic similarity, yet this is not the focus of our topic model. On the contrary, we explicitly allow for a comparatively larger diversity among headlines within the same topic. The reason is that our topic categorization should cover thematic areas (rather than specific news events) and should thus be comparatively broad.

## F.4 Regression analysis with topic controls

Using the above topics, we then repeated our main analysis while controlling for between-topic heterogeneity. Overall, the parameter estimates for the extended models are qualitatively similar for both positive and negative words (Supplementary Table 16). We find that, on average, the categories "Economy & Government", "Life", "LGBT", "Women Rights and Feminism" and "Parenting & School" attract fewer clicks than the reference category "Entertainment."

| | Coef | Lower CI | Upper CI | $P$-value |
|---|---|---|---|---|
| *Positive* | −0.008 | −0.010 | −0.006 | < 0.001 |
| *Negative* | 0.015 | 0.013 | 0.018 | < 0.001 |
| TOPICS | | | | |
| Entertainment (reference topic) | — | — | — | — |
| Government & Economy | −0.531 | −0.581 | −0.481 | < 0.001 |
| LGTB | −0.112 | −0.182 | −0.042 | < 0.001 |
| Life | −0.393 | −0.438 | −0.348 | < 0.001 |
| Parenting & School | −0.247 | −0.301 | −0.194 | < 0.001 |
| People | 0.006 | −0.060 | 0.072 | 0.808 |
| Women Rights & Feminism | −0.063 | −0.116 | −0.010 | 0.002 |
| CONTROL VARIABLES | | | | |
| *Length* | 0.040 | 0.038 | 0.043 | < 0.001 |
| *Complexity* | −0.004 | −0.006 | −0.001 | < 0.001 |
| *PlatformAge* | −0.316 | −0.330 | −0.303 | < 0.001 |
| (Intercept) | −4.212 | −4.250 | −4.175 | < 0.001 |

Observations: 53,699

Supplementary Table 16: Regression results estimating the effect of positive and negative words on the click-through rate. Here, dummy variables referring to the different topics are included. Reported are standardized coefficient estimates and $99\,\%$ CIs. $P$-values are calculated using two-sided $z$-tests. Experiment-specific intercepts (i. e., random effects) are included. $N = 53{,}669$ headlines were examined over $12{,}448$ RCTs.

## F.5 Regression analysis with topic-specific negativity effects

We further examine interactions between topics and emotional variables. Regression estimates show that the negative effects for positive and negative words found in the main analysis are also present for the majority of topics (Supplementary Table 17).

| | Coef | Lower CI | Upper CI | $P$-value |
|---|---|---|---|---|
| $Positive$ × Entertainment | 0.005 | −0.001 | 0.010 | 0.023 |
| $Positive$ × Government & Economy | −0.014 | −0.021 | −0.008 | < 0.001 |
| $Positive$ × LGTB | 0.002 | −0.007 | 0.011 | 0.505 |
| $Positive$ × Life | −0.011 | −0.015 | −0.007 | < 0.001 |
| $Positive$ × Parenting & School | −0.017 | −0.024 | −0.011 | < 0.001 |
| $Positive$ × People | −0.025 | −0.036 | −0.014 | < 0.001 |
| $Positive$ × Women Rights & Feminism | −0.004 | −0.010 | 0.002 | 0.093 |
| $Negative$ × Entertainment | 0.011 | 0.005 | 0.016 | < 0.001 |
| $Negative$ × Government & Economy | 0.029 | 0.024 | 0.035 | < 0.001 |
| $Negative$ × LGBT | −0.001 | −0.011 | 0.009 | 0.845 |
| $Negative$ × Life | 0.015 | 0.011 | 0.019 | < 0.001 |
| $Negative$ × Parenting & School | 0.018 | 0.011 | 0.025 | < 0.001 |
| $Negative$ × People | 0.008 | −0.002 | 0.017 | 0.036 |
| $Negative$ × Women's Rights & Feminism | 0.013 | 0.007 | 0.018 | < 0.001 |
| $Length$ | 0.041 | 0.038 | 0.043 | < 0.001 |
| $Complexity$ | −0.004 | −0.006 | −0.001 | < 0.001 |
| $PlatformAge$ | −0.309 | −0.323 | −0.295 | < 0.001 |
| (Intercept) | −4.476 | −4.490 | −4.461 | < 0.001 |

Observations: 53,699

Supplementary Table 17: Regression results estimating the effect of positive and negative words on the click-through rate. Here, we examine interactions between topic dummies and positive/negative words. Reported are standardized coefficient estimates and 99 % CIs. $P$-values are calculated using two-sided $z$-tests. Experiment-specific intercepts (i. e., random effects) are included. $N = 53{,}669$ headlines were examined over 12,448 RCTs.

# G Extension to discrete emotions

## G.1 Frequency of emotional words from NRC emotion lexicon

The most common emotional words from the NRC emotion lexicon (Supplementary Figure 7) are categorized as belonging to *trust*, for which the average relative proportion of all emotional words in headlines amounts to 23.5%. This is followed by *anticipation* (16.2%) and *joy* (14.4%). In contrast, emotional words belonging to *surprise* (6.3%) and *disgust* (6.7%) are less frequent.

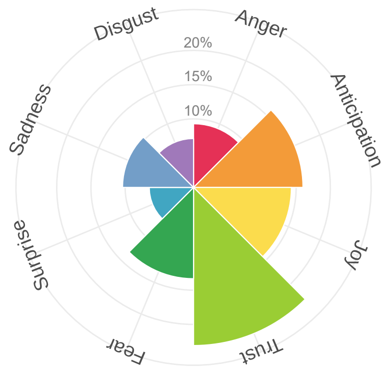


Supplementary Figure 7: Average relative proportion of emotional words in news headlines. Here, the categorization involves eight basic emotions as provided by the NRC emotion lexicon [1, 2].

A list of the most frequent emotional words from the NRC emotion lexicon is given Supplementary Table 18. Note that words that appear unexpected at a first glance are often used in a context that is characterized by a specific emotion. For example, the term “boy” is often part of the expression “Oh boy! . . . ” where it is used to signal strong opposition and even *disgust*. Similarly, the term “watch” was often used in the context of “watch out” where, as a result, the headline was perceived as communicating *fear*. For details on why specific words were classified by users in a large-scale study as embedding a specific emotion, we refer to the original paper developing the NRC emotion lexicon [1, 2].

| *Anger* | | *Anticipation* | | *Disgust* | | *Fear* | |
|---|---|---|---|---|---|---|---|
| Word | Frequency | Word | Frequency | Word | Frequency | Word | Frequency |
| money | 636 | time | 1772 | bad | 588 | watch | 1640 |
| words | 634 | watch | 1640 | awful | 363 | change | 913 |
| bad | 588 | pretty | 746 | powerful | 336 | bad | 588 |
| awful | 363 | money | 636 | boy | 320 | awful | 363 |
| powerful | 336 | white | 631 | hate | 300 | powerful | 336 |
| hate | 300 | sex | 447 | john | 267 | government | 322 |
| death | 258 | marriage | 414 | death | 258 | hate | 300 |
| homeless | 233 | happy | 356 | finally | 239 | war | 294 |
| fight | 229 | powerful | 336 | sick | 236 | death | 258 |
| hell | 213 | wait | 334 | homeless | 233 | homeless | 233 |
| *Joy* | | *Sadness* | | *Surprise* | | *Trust* | |
| Word | Frequency | Word | Frequency | Word | Frequency | Word | Frequency |
| love | 980 | black | 766 | money | 636 | real | 909 |
| pretty | 746 | bad | 588 | hilarious | 480 | school | 763 |
| beautiful | 645 | awful | 363 | guess | 339 | pretty | 746 |
| money | 636 | hate | 300 | death | 258 | money | 636 |
| white | 631 | music | 271 | hope | 248 | white | 631 |
| food | 568 | death | 258 | deal | 245 | food | 568 |
| hilarious | 480 | sick | 236 | finally | 239 | truth | 505 |
| sex | 447 | homeless | 233 | teach | 191 | word | 461 |
| save | 418 | die | 214 | leave | 183 | sex | 447 |
| marriage | 414 | hell | 213 | celebrity | 158 | secret | 427 |

Supplementary Table 18: Top 10 most frequent words for each of the 8 basic emotions, as defined by the NRC emotion lexicon, in our sample.

## G.2 Estimation results for discrete emotions

As secondary analyses, we study the role of discrete emotions from the NRC emotion lexicon. Detailed estimation results are reported in Supplementary Table 19. Here we focus on the four discrete emotions for which we found statistically significant positive correlation between the perceptions of emotions and the computed NRC emotion scores (i. e., *Anger*, *Fear*, *Joy*, *Sadness*).

| | Coef | Lower CI | Upper CI | $P$-value |
|---|---|---|---|---|
| $Anger$ | 0.000 | −0.003 | 0.002 | 0.666 |
| $Fear$ | −0.007 | −0.009 | −0.004 | $< 0.001$ |
| $Joy$ | −0.009 | −0.012 | −0.006 | $< 0.001$ |
| $Sadness$ | 0.006 | 0.003 | 0.009 | $< 0.001$ |
| $Length$ | 0.037 | 0.034 | 0.040 | $< 0.001$ |
| $Complexity$ | −0.004 | −0.007 | −0.001 | $< 0.001$ |
| $PlatformAge$ | −0.312 | −0.327 | −0.298 | $< 0.001$ |
| (Intercept) | −4.483 | −4.498 | −4.467 | $< 0.001$ |

Observations: 39,857

Supplementary Table 19: Regression model explaining click-through rate based on discrete emotions in headlines. Reported are standardized coefficient estimates and $99\,\%$ CIs. $P$-values are calculated using two-sided $z$-tests. Experiment-specific intercepts (i.e., random effects) are included. $N = 39{,}857$ headlines were examined over $11{,}934$ RCTs.

## G.3 Analysis with topics and emotions

Supplementary Table 20 controls for topic dummies in our regression estimating the effect of discrete emotions. Even when controlling for between-topic variation in clickability, the results remain robust.

| | Coef | Lower CI | Upper CI | $P$-value |
|---|---|---|---|---|
| DISCRETE EMOTIONS | | | | |
| *Anger* | 0.000 | −0.003 | 0.002 | 0.646 |
| *Fear* | −0.006 | −0.009 | −0.003 | < 0.001 |
| *Joy* | −0.009 | −0.013 | −0.006 | < 0.001 |
| *Sadness* | 0.006 | 0.003 | 0.008 | < 0.001 |
| TOPICS | | | | |
| Entertainment (reference topic) | — | — | — | — |
| Government & Economy | −0.536 | −0.588 | −0.484 | < 0.001 |
| LGTB | −0.115 | −0.188 | −0.041 | < 0.001 |
| Life | −0.398 | −0.445 | −0.351 | < 0.001 |
| Parenting & School | −0.245 | −0.301 | −0.189 | < 0.001 |
| People | 0.014 | −0.055 | 0.084 | 0.594 |
| Women Rights & Feminism | −0.074 | −0.130 | −0.019 | < 0.001 |
| CONTROL VARIABLES | | | | |
| *Length* | 0.038 | 0.035 | 0.041 | < 0.001 |
| *Complexity* | −0.004 | −0.007 | −0.001 | < 0.001 |
| *PlatformAge* | −0.320 | −0.334 | −0.306 | < 0.001 |
| (Intercept) | −4.221 | −4.260 | −4.182 | < 0.001 |

Observations: 39,857

Supplementary Table 20: Regression results estimating the effect of discrete emotions on the click-through rate. Here, dummy variables referring to the different topics are included. Reported are standardized coefficient estimates and $99\,\%$ CIs. $P$-values are calculated using two-sided $z$-tests. Experiment-specific intercepts (i. e., random effects) are included. $N = 39{,}857$ headlines were examined over $11{,}934$ RCTs.

# H User studies to validate dictionary approach

In line with best practices [3], we re-validated both the LIWC dictionary and the NRC emotion lexicon for our setting. For this, we conducted two user studies during Stage 1 using the exploratory sample from the Upworthy Research Archive.

*User study 1*

In user study 1, we validated that user judgments of positivity/negativity and our computed ratings of sentiment from the LIWC dictionary were significantly correlated. Participants were recruited from the New York University subject pool, provided informed consent, and were granted .5 research credit hours for their participation. Participants viewed a total of 213 headlines drawn randomly from the exploratory dataset. All participants were native English speakers. To avoid fatigue, participants and headlines were split into two groups, so that each had to respond to only a subset of all questions. We recruited two groups of $k = 10$ participants; after removing participants who failed to complete the study, we were left with one group of 8 raters, and one group of 10 raters, a standard number of raters for validations in prior literature [3]. The number of headlines ($N = 213$) was chosen based on best practices [3]. Specifically, 50 RCTs were randomly selected from the 2,602 RCTs in our filtered preliminary sample. All headlines in an RCT package were included for a total of 213 headlines to be tested. No statistical methods were used to pre-determine sample sizes but our sample sizes are similar to those reported in previous publications [3]. For each headline, participants were asked *"How negative or positive is this headline?"* Participants rated each headline on a $-3$ (very negative) to $+3$ (very positive) Likert scale. We refer to the score as "sentiment" in the following.

We first assessed the inter-rater agreement using Kendall's $W$. The inter-rater agreement was statistically significant ($W = 0.33$, $p < 0.001$).

Both user ratings and dictionary scores (as used in our main analysis) are not directly comparable. The reason is that user ratings refer to an *overall* sentiment (on a scale from negative to

positive), whereas the independent variables are two *separate* scores for positivity and negativity. Hence, we show that both ratings and dictionary scores are related in the following ways:

- We separately compare the sentiment rating with the positivity and negativity scores (i. e., $Positive$ and $Negative$, respectively). Reassuringly, we use the same dictionary approach as in the main paper, including negation handling. The statistical comparison is based on Spearman's rank correlation coefficient ($r_s$). For positivity, the correlation is $r_s = 0.20$ and statistically significant ($p = 0.004$). For negativity, the correlation is $r_s = -0.20$ and statistically significant ($p = 0.003$). Hence, changes in the proportion of positive and negative words in a headline are also reflected in the perceived sentiment of raters.

- We map the two separate dictionary scores onto a combined sentiment score. For this, we compute the net difference between positivity and negativity in the text (i. e., $Sentiment = Positive - Negative$). We then compare the sentiment ratings against the dictionary-based sentiment scores. Specifically, we compute Spearman's rank correlation coefficient ($r_s$) between the mean rantings of the 8 human judges' scores with the dictionary scores. User ratings of sentiment and computed sentiment scores were moderately but significantly correlated with one another ($r_s = 0.30$, $p < 0.001$).

Altogether, this validates that, for our news headlines, user perceptions of negativity and computed negativity scores are related. Importantly, this result also confirms that dictionary words are subject to additivity, that is, that a headline that includes two negative words is perceived as being more negative than a headline that includes only one negative word.

*User study 2*

In user study 2, we validated that user judgments of discrete emotion and our computed emotion scores from the NRC emotion lexicon were significantly correlated. Again, participants were recruited from the NYU subject pool, were native English speakers, provided informed consent, and were granted .5 research credit hours for their participation. Participants viewed a total of

213 headlines drawn randomly from the exploratory dataset. To avoid fatigue, four groups of participants were recruited, so that each had to respond to only a subset of questions. The number of headlines ($N = 213$) was again chosen based on best practices [3]. One participant was removed for failing to complete the study, leaving three groups of 10 raters, and one group of 9 raters. Again, no statistical methods were used to pre-determine sample sizes but our sample sizes are similar to those reported in previous publications [3]. For each headline, participants were asked *"How much _____ is present in this headline?"* The blank space in the question was repeatedly replaced by all of the 8 basic emotions from the NRC emotion lexicon (i. e., $Anticipation$, $Disgust$, $Fear$, $Joy$, $Sadness$, $Surprise$, $Trust$). This corresponds to $213 \times 8 = 1704$ questions. For each headline, participants gave ratings for all 8 emotions on a 1 (no _____) to 7 (a great deal of _____) Likert scale.

The inter-rater agreement is listed in Supplementary Table 21. It was statistically significant for 7 of the discrete emotions ($Anger$, $Anticipation$, $Disgust$, $Fear$, $Sadness$, and $Disgust$).

We found that the overall correlation between NRC dictionary scores and the mean ratings of the user judgments for the 8 discrete emotions was positive and statistically significant ($r_s = 0.11$; $p < 0.001$). The correlations for the mean user user ratings of each emotion and the computed emotion score are presented in Supplementary Table 22. For specific emotions, user judgments for $Anger$, $Fear$, $Joy$, and $Sadness$ were significantly correlated with the computed emotion scores. For these emotions, the results validate that emotion ratings from users and NRC dictionary scores are, to a large extent, meaningfully related.

In our regression analysis, we focus the four discrete emotions for which we found statistically significant positive correlation between the perceptions of emotions and the computed NRC emotion scores (i. e., *Anger*, *Fear*, *Joy*, *Sadness*). For thoroughness, we also analyze the effects of all other discrete emotions (i. e., $Anticipation$, $Disgust$, $Surprise$, $Trust$) in Supplement I.

| Emotion | Kendall's $W$ | $P$-value |
|---|---|---|
| *Sentiment* | 0.33 | $< 0.001$ |
| *Anger* | 0.24 | $< 0.001$ |
| *Anticipation* | 0.17 | $< 0.001$ |
| *Disgust* | 0.22 | $< 0.001$ |
| *Fear* | 0.23 | $< 0.001$ |
| *Joy* | 0.15 | 0.008 |
| *Sadness* | 0.23 | $< 0.001$ |
| *Surprise* | 0.13 | 0.071 |
| *Trust* | 0.19 | $< 0.001$ |

Supplementary Table 21: Kendall's $W$ coefficient for the inter-rater agreement between users. $P$-values are calculated using two-sided Chi-square tests.

| Emotion | Correlation | $P$-value |
|---|---|---|
| *Anger* | 0.22 | 0.005 |
| *Anticipation* | $-0.07$ | 0.341 |
| *Disgust* | 0.01 | 0.926 |
| *Fear* | 0.29 | $< 0.001$ |
| *Joy* | 0.30 | 0.002 |
| *Sadness* | 0.30 | $< 0.001$ |
| *Surprise* | $-0.06$ | 0.414 |
| *Trust* | 0.12 | 0.122 |

Supplementary Table 22: Spearman's rank correlation coefficient ($r_s$) between user judgments and dictionary scores for emotional words. $P$-values are calculated using two-sided Spearman's tests.

# I Analysis across all basic emotions and higher-order emotions

## I.1 Analysis for basic emotions

In our main regression analysis, we focused on 4 discrete emotions (i. e., *anger*, *fear*, *joy*, *sadness*) for which we found a notable correlation between the computed NRC emotion scores and human judgments, implying that humans perceive a headline to embed that emotions. For thoroughness, we performed a regression analysis based on all 8 basic emotions from the NRC emotion lexicon. This should be interpreted with caution, as humans do not necessarily read the same emotions in the headlines, and thus they should understood as "NRC dimensions."

Of note, the variables for the 8 basic emotions sum to 1 and are thus subject to linear dependency. Evidently, there are high cross-correlations among the 8 basic emotions (see Supplementary Figure 8). Methodologically, they are relevant because they prohibit all 8 emotions to be examined in the same model without making the model rank deficient. To alleviate issues due to linear dependence, we performed a regression analysis based on 8 separate regression models that were estimated independently, each including one of the 8 basic emotions. The multilevel regression for the 8 basic emotions is specified analogous to our analysis from the main paper, i. e.,

$$\text{logit}(\theta_{ij}) = \alpha + \alpha_i + \beta\, \mathit{BasicEmotion}_{ij} + \gamma_1\, \mathit{Length}_{ij} + \gamma_2\, \mathit{Complexity}_{ij} + \gamma_3\, \mathit{PlatformAge}_{ij} \tag{1}$$

with a random effects specification, where $\alpha$ is the global intercept and $\alpha_i$ captures the heterogeneity among experiments $i = 1, \ldots, N$. Further, $\mathit{BasicEmotion}_{ij}$ denotes one of the 8 basic emotions (e. g., $\mathit{Anger}_{ij}$, $\mathit{Anticipation}_{ij}$, etc.). In addition, we again control for length, text complexity, and the age of the platform since the first overall experiment. The coefficient $\beta$ then quantifies how one of the basic emotions affects the click-through rate. To account for multiple hypothesis testing, we use Bonferroni correction [12].

The estimation results confirm the findings from the main analysis (Supplementary Table 23

and Supplementary Figure 9). As in the main paper, positive effects are found for *sadness* and *disgust*. In addition, a statistically significant negative effect is found for *joy* and *fear*. The effect of *surprise* is statistically significant at the 1 % level, but does not survive Bonferroni correction. Due to the estimation procedure, we refrain from comparing the effect size of the different basic emotions.

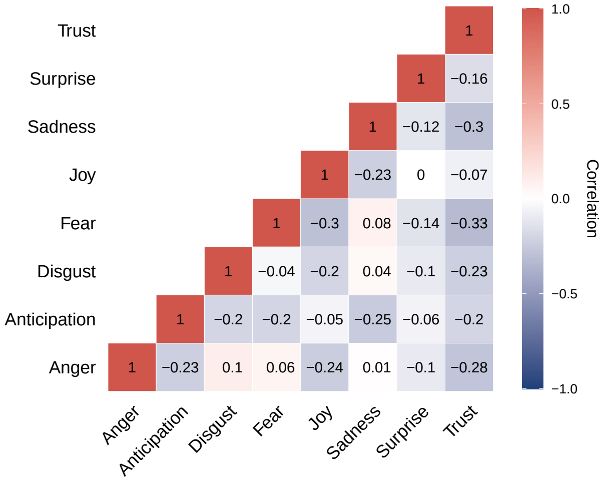


Supplementary Figure 8: Cross-correlations between variables representing emotional words in news headlines. Here, the emotional variables are the proportion of emotional words as defined by NRC emotion lexicon. Pearson's correlation coefficients are reported.

|  | Coef | Lower CI | Upper CI | $P$-value |
|---|---|---|---|---|
| $Anger$ | 0.001 | −0.002 | 0.005 | 0.2 |
| $Anticipation$ | 0.000 | −0.003 | 0.004 | 0.852 |
| $Disgust$ | 0.005 | 0.002 | 0.009 | < 0.001 |
| $Fear$ | −0.004 | −0.007 | 0.000 | < 0.001 |
| $Joy$ | −0.009 | −0.012 | −0.005 | < 0.001 |
| $Sadness$ | 0.007 | 0.004 | 0.011 | < 0.001 |
| $Surprise$ | 0.003 | 0.000 | 0.006 | 0.005 |
| $Trust$ | −0.002 | −0.005 | 0.002 | 0.152 |

Observations: 39,857

Supplementary Table 23: Estimation results for the model with all basic emotions. Coefficients are retrieved from separate models for each dyad pair due to linear dependence between the dyads. Reported are standardized coefficient estimates and $99\,\%$ CIs. $P$-values are calculated using two-sided $z$-tests and Bonferroni-corrected [12]. Experiment-specific intercepts (i. e., random effects) are included. $N = 39{,}857$ headlines were examined over $11{,}934$ RCTs.

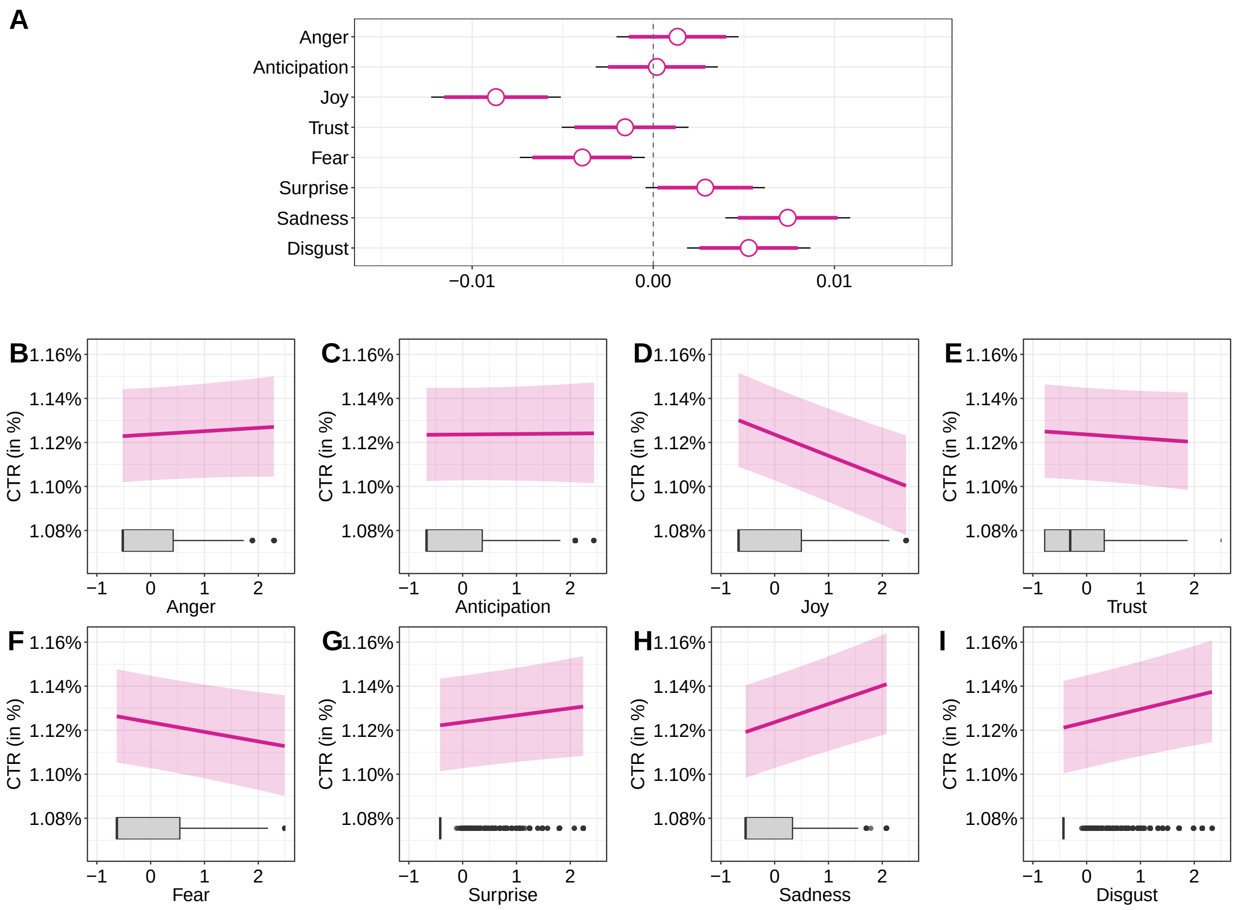


Supplementary Figure 9: Effect of emotional words in news headlines on the click-through rate. $N = 39{,}857$ headlines were examined over $11{,}934$ RCTs. **(A)** Shown are the estimates of the standardized coefficient (circles) that originate from separate regressions for the basic emotions as derived from the NRC emotion lexicon. The thick (pink) and thin (black) error bars correspond to the 99 % confidence intervals (CIs) and 99 % Bonferroni-corrected [12] CIs, respectively. **(B-I)**: Predicted marginal effects of basic emotions on the click-through rate (lines). The error bands (shaded area) correspond to the 99 % confidence intervals (CIs). Boxplots show the distribution of the variables in our sample (center line gives the median; box limits are upper and lower quartiles; whiskers denote minimum/maximum; points are outliers defined as being beyond 1.5x of the interquartile range). Full estimation results are in Supplementary Table 23.

## I.2 Analysis for bipolar emotion pairs

Following [13, 14], we analyzed the effects of bipolar emotion pairs on the click-through rate. Specifically, we arranged the basic emotions into 4 pairs of bipolar emotions (i. e., so that they

represent opposite petals as in Plutchik's model [15]). The 4 bipolar emotions are *anticipation–surprise*, *anger–fear*, *trust–disgust*, and *joy–sadness*, representing the pairs of emotions that are least similar to one another. The corresponding variables for the bipolar emotions are computed by taking the difference between the two (thereby yielding a value between $-1$ and 1). This yields 4 scores: $\mathit{AnticipationSurprise}_{ij} = \mathit{Anticipation}_{ij} - \mathit{Surprise}_{ij}$, $\mathit{AngerFear}_{ij} = \mathit{Anger}_{ij} - \mathit{Fear}_{ij}$, $\mathit{TrustDisgust}_{ij} = \mathit{Trust}_{ij} - \mathit{Disgust}_{ij}$, and $\mathit{JoySadness}_{ij} = \mathit{Joy}_{ij} - \mathit{Sadness}_{ij}$.

The multilevel regression is specified analogous the previous models but with additional explanatory variables, i. e.,

$$\begin{aligned} \operatorname{logit}(\theta_{ij}) = \alpha + \alpha_i + \beta_1\, \mathit{AngerFear}_{ij} + \beta_2\, \mathit{AnticipationSurprise}_{ij} + \beta_3\, \mathit{JoySadness}_{ij} \\ + \beta_4\, \mathit{TrustDisgust}_{ij} + \gamma_1\, \mathit{Length}_{ij} + \gamma_2\, \mathit{Complexity}_{ij} \end{aligned} \tag{2}$$

where $\alpha$ and $\alpha_i$ represent the varying-intercept specification. Specifically, $\alpha$ is again the global intercept and $\alpha_i$ captures the heterogeneity across experiments $i = 1, \ldots, N$. As in the main paper, we include the control variables, i. e., length and text complexity. The coefficients $\beta_1, \ldots, \beta_4$ quantify the effect of the four bipolar emotion pairs (i. e., *anticipation–surprise*, *anger–fear*, *trust–disgust*, and *joy–sadness*) on the click-through rate.

We found negative coefficients for words from the bipolar emotion pairs *joy–sadness* (coef: $-0.010$, SE = 0.001, $z = -8.908$, $p < 0.001$, CI = $[-0.013, -0.007]$) and *trust–disgust* (coef: $-0.003$, SE = 0.001, $z = -2.312$, $p = 0.021$, CI = $[-0.005, -0.0003]$). We also found a positive coefficient for the bipolar emotion pair *anger–fear* (coef: $0.005$, SE = 0.001, $z = 4.771$, $p < 0.001$, CI = $[0.002, 0.008]$). The negative signs imply that a higher click-through rate is elicited by headlines containing a greater proportion of words belonging to *sadness*, *disgust*, and *surprise* (Supplementary Table 24 and Supplementary Figure 10). The coefficient estimates for the pair *anticipation–surprise* was not statistically significant at common significance thresholds. Consistent with our previous findings, we observed that the click-through rate increases as the text length increases and text complexity scores decrease. Again, the click-through rate was lower for

headlines at the end of Upworthy's career.

For thoroughness, we also analyzed emotions for which we did not found statistically significant positive correlation between the user judgments and the computed NRC emotion scores in the validation study.

| | Coef | Lower CI | Upper CI | $P$-value |
|---|---|---|---|---|
| $\mathit{AngerFear}$ | 0.005 | 0.002 | 0.008 | $< 0.001$ |
| $\mathit{AnticipationSurprise}$ | $-0.001$ | $-0.004$ | 0.002 | 0.282 |
| $\mathit{TrustDisgust}$ | $-0.003$ | $-0.005$ | 0.000 | 0.021 |
| $\mathit{JoySadness}$ | $-0.010$ | $-0.013$ | $-0.007$ | $< 0.001$ |
| $\mathit{Length}$ | 0.037 | 0.034 | 0.040 | $< 0.001$ |
| $\mathit{Complexity}$ | $-0.004$ | $-0.007$ | $-0.001$ | $< 0.001$ |
| $\mathit{PlatformAge}$ | $-0.312$ | $-0.327$ | $-0.297$ | $< 0.001$ |
| (Intercept) | $-4.482$ | $-4.498$ | $-4.467$ | $< 0.001$ |

Observations: 39,857

Supplementary Table 24: Regression model explaining click-through rate based on bipolar emotion pairs in headlines. Reported are standardized coefficient estimates and $99\,\%$ CIs. $P$-values are calculated using two-sided $z$-tests. Experiment-specific intercepts (i. e., random effects) are included. $N = 39{,}857$ headlines were examined over $11{,}934$ RCTs.

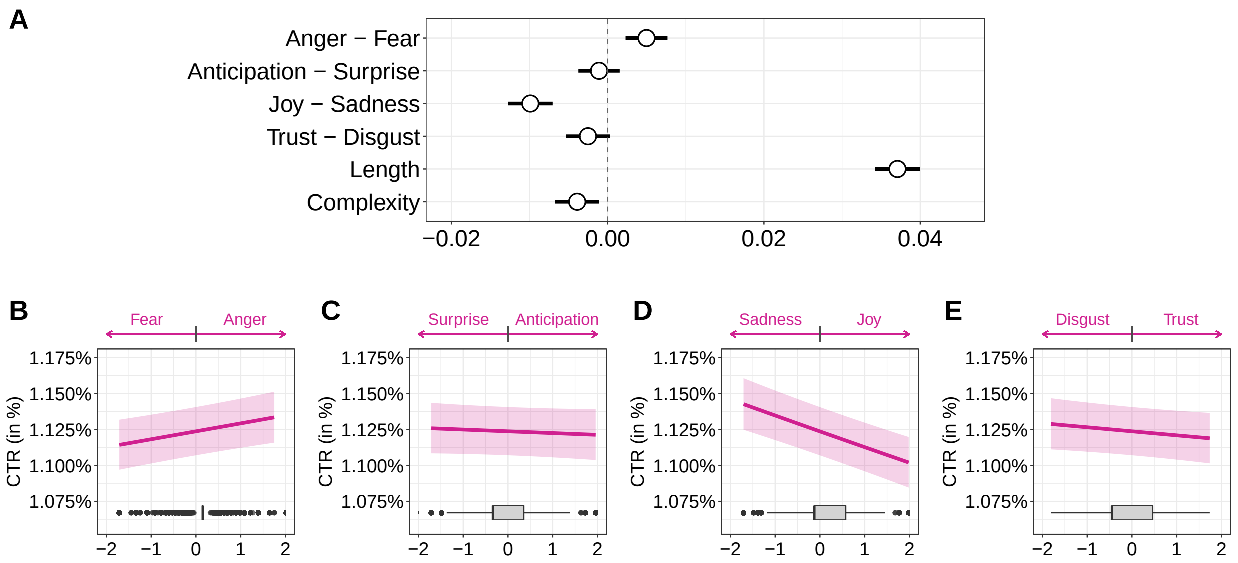


Supplementary Figure 10: Effect of emotional words on the click-through rate. $N = 39{,}857$ headlines were examined over $11{,}934$ RCTs. (A) Shown are the estimated standardized coefficients (circles) and 99% confidence intervals (error bars) for each bipolar emotion derived from the NRC emotion lexicon. Overall, clicks are elicited by news headlines with words classified as *surprise* and *sadness*. The variable *PlatformAge* is included in the model during estimation but not shown for better readability. (B-E) Predicted marginal effects of bipolar emotions on the click-through rate (lines). The error bands (shaded area) correspond to the 99 % confidence intervals (CIs). In (B), the boxplots indicate narrow distribution for the *fear–anger* pair, suggesting that the variation in these emotions is comparatively small. Boxplots show the distribution of the variables in our sample (center line gives the median; box limits are upper and lower quartiles; whiskers denote minimum/maximum; points are outliers defined as being beyond 1.5x of the interquartile range). Full estimation results are in Supplementary Table 24.

## I.3 Analysis for emotional dyads

Plutchik's emotions model defines 24 emotional dyads, which are more complex emotions composed of two basic emotions [15]. Following [13, 14], we compute the score for each of the 24 emotional dyads by taking the sum of two emotions (e. g., $Aggressiveness_{ij} = Anger_{ij} + Anticipation_{ij}$). Then, we will compute a score for each of the opposite pairs by taking the corresponding difference (e. g., $LoveRemorse_{it} = Love_{it} - Remorse_{it}$). Across all dyads, this will yield 12 different scores to be used in a regression analysis.

We examine the effect of emotional dyads on the click-through rate as follows. We fit twelve separate models, that is, one for each pair among the emotional dyads, due to linear dependencies between the dyads. The underlying model is given by

$$\text{logit}(\theta_{ij}) = \alpha + \alpha_i + \beta\, \mathit{EmotionalDyad}_{ij} + \gamma_1\, \mathit{Length}_{ij} + \gamma_2\, \mathit{Complexity}_{ij}, \tag{3}$$

with global intercept $\alpha$ and varying intercept $\alpha_i$ and $\mathit{EmotionalDyads}_{ij}$ denotes one pair among the emotional dyads. We include the same control variables as in the previous models. To account for multiple hypothesis testing, we again use Bonferroni correction [12].

The regression results (Supplementary Table 25 and Supplementary Figure 11) show a negative coefficient for emotional words from the following dyads: *optimism–disapproval*, *love–remorse*, *submission–contempt*, *hope–unbelief*, *guilt–envy*, *anxiety–outrage*, and *delight–pessimism*. Users thus have a propensity to respond to language expressing *disapproval*, *remorse*, *contempt*, *unbelief*, *envy*, *outrage*, and *pessimism*, whereas the click-through rate decreases due to the presence of *optimism*, *love*, *submission*, *hope*, *guilt*, *anxiety*, and *delight*, There was one dyad with a positive coefficient: *despair–pride*, meaning users have a propensity to respond more to language related to *despair* and less to language related to *pride*. Overall, we found that several dyads are important determinants of click-through rates.

| | Coef | Lower CI | Upper CI | $P$-value |
|---|---|---|---|---|
| $OptimismDisapproval$ | $-0.008$ | $-0.012$ | $-0.005$ | $< 0.001$ |
| $LoveRemorse$ | $-0.009$ | $-0.013$ | $-0.005$ | $< 0.001$ |
| $SubmissionContempt$ | $-0.006$ | $-0.009$ | $-0.002$ | $< 0.001$ |
| $AweAggressiveness$ | $-0.002$ | $-0.005$ | $0.002$ | 0.105 |
| $HopeUnbelief$ | $-0.004$ | $-0.008$ | $-0.001$ | $< 0.001$ |
| $GuiltEnvy$ | $-0.011$ | $-0.014$ | $-0.007$ | $< 0.001$ |
| $CuriosityCynicism$ | $-0.002$ | $-0.005$ | $0.002$ | 0.07 |
| $DespairPride$ | $0.004$ | $0.001$ | $0.008$ | $< 0.001$ |
| $AnxietyOutrage$ | $-0.004$ | $-0.008$ | $-0.001$ | $< 0.001$ |
| $DelightPessimism$ | $-0.006$ | $-0.009$ | $-0.002$ | $< 0.001$ |
| $SentimentalityMorbidness$ | $0.002$ | $-0.002$ | $0.005$ | 0.079 |
| $ShameDominance$ | $0.000$ | $-0.003$ | $0.004$ | 0.64 |

Observations: $39{,}857$

Supplementary Table 25: Estimation results for the model with emotional dyads. Coefficients are retrieved from separate models for each dyad pair due to linear dependence between the dyads. Reported are standardized coefficient estimates and $99\,\%$ CIs. $P$-values are calculated using two-sided $z$-tests and Bonferroni-corrected [12]. Experiment-specific intercepts (i. e., random effects) are included. $N = 39{,}857$ headlines were examined over $11{,}934$ RCTs.

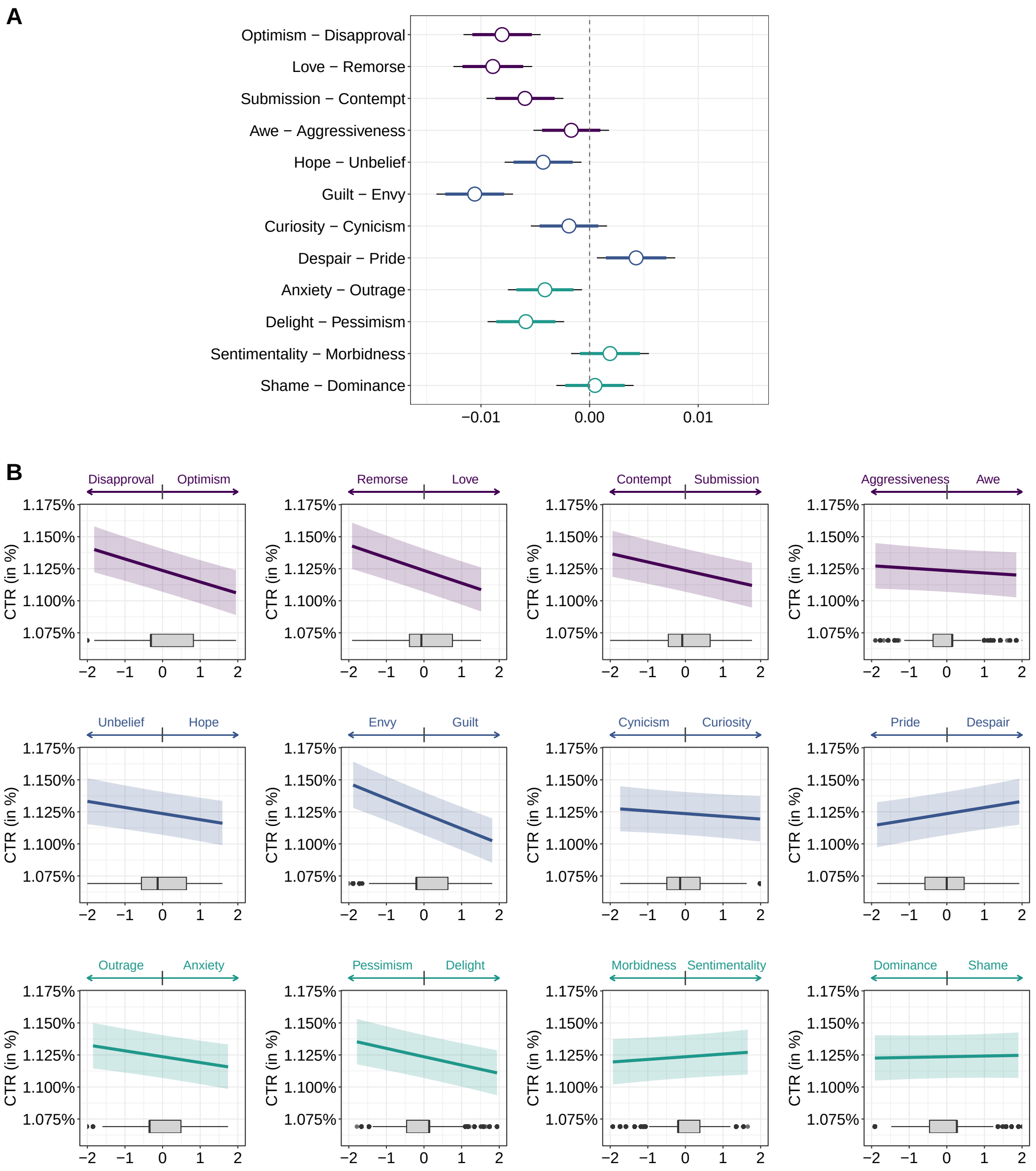


Supplementary Figure 11: Effect of emotional words from emotional dyads on the click-through rate. $N = 39{,}857$ headlines were examined over $11{,}934$ RCTs. (A) Shown are the estimated standardized coefficients (circles) for emotional dyads. The thick (colored) and thin (black) error bars correspond to 99% confidence intervals and Bonferroni-corrected 99% confidence intervals, respectively. Due to linear dependencies among the dyads, the estimates originate from separate regressions. (B) Predicted marginal effects of the emotional words from the different dyads on the click-through rate (lines). The error bands (shaded area) correspond to the 99 % confidence intervals (CIs). The plots are arranged by primary (top), secondary (middle), and tertiary (bottom) dyads. Boxplots show the distribution of the variables in our sample (center line gives the median; box limits are upper and lower quartiles; whiskers denote minimum/maximum; points are outliers defined as being beyond 1.5x of the interquartile range). Full estimation results are in Supplementary Table 25.

# References

[1] Mohammad, S. & Turney, P. Emotions evoked by common words and phrases: Using mechanical turk to create an emotion lexicon. In *NAACL HLT 2010 Workshop on Computational Approaches to Analysis and Generation of Emotion in Text,* (2010).

[2] Mohammad, S. M. Sentiment analysis: Detecting valence, emotions, and other affectual states from text. In *Emotion Measurement*, 201–237 (2016).

[3] Song, H. *et al.* In validations we trust? The impact of imperfect human annotations as a gold standard on the quality of validation of automated content analysis. *Political Communication* **37**, 550–572 (2020).

[4] Pennebaker, J. W., Booth, R. J., Boyd, R. L. & Francis, M. E. Linguistic Inquiry and Word Count: LIWC2015 (2015). URL www.liwc.net.

[5] Thelwall, M., Buckley, K., Paltoglou, G., Di Cai & Kappas, A. Sentiment strength detection in short informal text. *Journal of the American Society for Information Science and Technology* **61**, 2544–2558 (2010).

[6] Brady, W. J., Wills, J. A., Jost, J. T., Tucker, J. A. & van Bavel, J. J. Emotion shapes the diffusion of moralized content in social networks. *Proceedings of the National Academy of Sciences (PNAS)* **114**, 7313–7318 (2017).

[7] Graham, J., Haidt, J. & Nosek, B. A. Liberals and conservatives rely on different sets of moral foundations. *Journal of Personality and Social Psychology* **96**, 1029–1046 (2009).

[8] Curiskis, S. A., Drake, B., Osborn, T. R. & Kennedy, P. J. An evaluation of document clustering and topic modelling in two online social networks: Twitter and Reddit. *Information Processing & Management* **57**, 102034 (2020).

[9] Cer, D. *et al.* Universal sentence encoder. URL https://arxiv.org/pdf/1803.11175.pdf.

[10] Toetzke, M., Banholzer, N. & Feuerriegel, S. Monitoring global development aid with machine learning. *Nature Sustainability* (2022).

[11] Chang, J., Gerrish, S., Wang, C., Boyd-Graber, J. L. & Blei, D. M. Reading tea leaves: How humans interpret topic models. In *Advances in Neural Information Processing Systems* (2009).

[12] Dunn, O. J. Multiple comparisons among means. *Journal of the American Statistical Association* **56**, 52–64 (1961).

[13] Pröllochs, N., Bär, D. & Feuerriegel, S. Emotions in online rumor diffusion. *EPJ Data Science* **10** (2021).

[14] Pröllochs, N., Bär, D. & Feuerriegel, S. Emotions explain differences in the diffusion of true vs. false social media rumors. *Scientific Reports* **11** (2021).

[15] Plutchik, R. *Emotion: Theory, research, and experience* (Academic Press, Orlando, 1984), 2 edn.
