# Supplementary material for: Negativity drives online news consumption
Source: Nat Hum Behav. 2023 Mar 16;7(5):812–22. doi: 10.1038/s41562-023-01538-4 (PMC10202797; doi:10.1038/s41562-023-01538-4)
Supplement: Supplementary file 2 — Reporting Summary [file 41562_2023_1538_MOESM2_ESM.pdf]

nature portfolio

Corresponding author(s): Jay J Van Bavel & Stefan Feuerriegel

Last updated by author(s): Jan 6, 2023

Reporting Summary

Nature Portfolio wishes to improve the reproducibility of the work that we publish. This form provides structure for consistency and transparency in reporting. For further information on Nature Portfolio policies, see our Editorial Policies and the Editorial Policy Checklist.

Statistics

For all statistical analyses, confirm that the following items are present in the figure legend, table legend, main text, or Methods section.

n/a	Confirmed	
☐	☒	The exact sample size (n) for each experimental group/condition, given as a discrete number and unit of measurement
☐	☒	A statement on whether measurements were taken from distinct samples or whether the same sample was measured repeatedly
☐	☒	The statistical test(s) used AND whether they are one- or two-sided<br>*Only common tests should be described solely by name; describe more complex techniques in the Methods section.*
☐	☒	A description of all covariates tested
☐	☒	A description of any assumptions or corrections, such as tests of normality and adjustment for multiple comparisons
☐	☒	A full description of the statistical parameters including central tendency (e.g. means) or other basic estimates (e.g. regression coefficient) AND variation (e.g. standard deviation) or associated estimates of uncertainty (e.g. confidence intervals)
☐	☒	For null hypothesis testing, the test statistic (e.g. F, t, r) with confidence intervals, effect sizes, degrees of freedom and P value noted<br>*Give P values as exact values whenever suitable.*
☒	☐	For Bayesian analysis, information on the choice of priors and Markov chain Monte Carlo settings
☒	☐	For hierarchical and complex designs, identification of the appropriate level for tests and full reporting of outcomes
☐	☒	Estimates of effect sizes (e.g. Cohen's d, Pearson's r), indicating how they were calculated

Our web collection on statistics for biologists contains articles on many of the points above.

Software and code

Policy information about availability of computer code

Data collection	The authors did not use any software for data collection, as the Upworthy Research Archive is an existing data set.
Data analysis	R version 4.0.2, including packages quanteda version 2.0.1, sentimentr version 2.7.1, and lme4 version 1.1.23 - all code is available at https://osf.io/uscpf/

For manuscripts utilizing custom algorithms or software that are central to the research but not yet described in published literature, software must be made available to editors and reviewers. We strongly encourage code deposition in a community repository (e.g. GitHub). See the Nature Portfolio guidelines for submitting code & software for further information.

Data

Policy information about availability of data

All manuscripts must include a data availability statement. This statement should provide the following information, where applicable:

- Accession codes, unique identifiers, or web links for publicly available datasets
- A description of any restrictions on data availability
- For clinical datasets or third party data, please ensure that the statement adheres to our policy

Both the exploratory and confirmatory data sets from Upworthy.com can be accessed here: https://osf.io/jd64p/. These data are from a 3rd party, we did not collect them ourselves, and they have been published before in Nature Scientific Data. The ciitation for the published data set is:
Matias, J. N., Munger, K., Le Quere, M. A., & Ebersole, C. (2021). The Upworthy Research Archive, a time series of 32,487 experiments in US media. Scientific Data,

8(1), 1-6. https://doi.org/10.1038/s41597-021-00934-7
Data for validation studies is available here: https://osf.io/uscpf/.
We used LIWC dictionary 2015. LIWC dictionaries are available for purchase at the following link: https://www.liwc.app/.

Human research participants

Policy information about studies involving human research participants and Sex and Gender in Research.

Reporting on sex and gender	Demographic information from validation participants was not collected, so we cannot report on the sex and gender makeup of the validation sample.
Population characteristics	Participants were New York University Undergraduate students who participated for .5 hours of course credit. Participants were fluent in English. No other demographic information was collected.
Recruitment	Participants were recruited from the New York University Student Subject Pool on SONA, and participated for .5 hours of course credit. Participants were all undergraduates and New York University who spoke fluent English, and no other demographic information was collected.
Ethics oversight	Institutional Review Board at New York University, Protocol IRB-FY2021-5555 and Institutional Review Board at ETH Zurich, Protocol 2020-N-151

Note that full information on the approval of the study protocol must also be provided in the manuscript.

Field-specific reporting

Please select the one below that is the best fit for your research. If you are not sure, read the appropriate sections before making your selection.

☐ Life sciences ☒ Behavioural & social sciences ☐ Ecological, evolutionary & environmental sciences

For a reference copy of the document with all sections, see nature.com/documents/nr-reporting-summary-flat.pdf

Behavioural & social sciences study design

All studies must disclose on these points even when the disclosure is negative.

Study description	Quantitative Experimental
Research sample	The research sample consisted of Upworthy.com Users. All data are aggregated at the headline level to ensure user privacy, so there is no demographic data for individual users included in the data set. Upworthy.com recorded impressions and clicks from people who visited their website. Thus, participants needed to be comfortable navigating the Internet. People who did not have internet access from 2013-2015 could not be included in our sample. We chose to use this sample because the data represented real-world behavior (i.e. clicking) and was experimentally controlled (due to Upworthy's use of RCTs.). N = 538,272,878 participant assignments. See Matias, J. N., Munger, K., Le Quere, M. A., & Ebersole, C. (2021). The Upworthy Research Archive, a time series of 32,487 experiments in US media. Scientific Data, 8(1), 1-6. for more details.<br><br>For the validation study, participants were recruited from the New York University Student Subject Pool on SONA, and participated for .5 hours of course credit. Participants were all undergraduates and New York University who spoke fluent English, and no other demographic information was collected. This sample is not representative due to it consisting of university students. We chose to use New York University undergraduates because they are a convenience sample.
Sampling strategy	Participants were sampled from people who navigated to Upworthy.com between 2013 and 2015, thus was a convenience sample. Power analysis in stage 1 revealed we have greater than 99% power to detect an effect size of 0.01 in the final sample.
Data collection	Data was collected from Upworthy.com from 2013-2015 by the staff at Upworthy.com. Because our analyses are on archival data, the people who collected the data were blind to our hypotheses. The data were collected passively, such that no experimenter was ever present to a participant, nor was anyone else.<br>When a person navigated to Upworthy.com, they were randomly assigned to see one possible story variation from a series of possible variations. Upworthy.com tested images, headlines, and lede text, but our analyses focus on headlines only. When a person sued a computer mouse or a touch screen to select to read an article, Upworthy.com recorded it as a click.
Timing	1-24-13 to 4-30-15
Data exclusions	Only RCTs where headlines were varied were used. Final sample was N = 11,109 headlines
Non-participation	Not Applicable. The data were collected passively and recored regular web browsing activity.
Randomization	Participants were randomly assigned to see one headline from a possible set in a given RCT when they navigated to Upworthy.com

Randomization	To achieve randomization, Upworthy used Ruby language (versions 2.9.3 through 2.3.1) and the RandomSample Method.

Reporting for specific materials, systems and methods

We require information from authors about some types of materials, experimental systems and methods used in many studies. Here, indicate whether each material, system or method listed is relevant to your study. If you are not sure if a list item applies to your research, read the appropriate section before selecting a response.

Materials & experimental systems

n/a	Involved in the study
☒	☐ Antibodies
☒	☐ Eukaryotic cell lines
☒	☐ Palaeontology and archaeology
☒	☐ Animals and other organisms
☒	☐ Clinical data
☒	☐ Dual use research of concern

Methods

n/a	Involved in the study
☒	☐ ChIP-seq
☒	☐ Flow cytometry
☒	☐ MRI-based neuroimaging
